# Thymol turbidity test is associated with the risk of cyclops syndrome following anterior cruciate ligament reconstruction

**DOI:** 10.1186/s12891-018-2286-1

**Published:** 2018-10-12

**Authors:** Yuya Kodama, Takayuki Furumatsu, Tomohito Hino, Yusuke Kamatsuki, Yoshiki Okazaki, Shin Masuda, Yuki Okazaki, Toshifumi Ozaki

**Affiliations:** Department of Orthopaedic Surgery, Okayama University Graduate School of Medicine, Dentistry, and Pharmaceutical Sciences, 2-5-1 Shikatacho, Kitaku, Okayama, 700-8558 Japan

**Keywords:** Cyclops syndrome, Anterior cruciate ligament, Thymol turbidity test, Cyclops nodule, Knee extension, Range of motion, Cyclopoid scar

## Abstract

**Background:**

Cyclops nodule formation is a serious complication after anterior cruciate ligament (ACL) reconstruction. The purpose of our study was to investigate whether an increase in thymol turbidity test (TTT) values is involved in the development of cyclops nodule formation or cyclopoid scar formation following ACL reconstruction.

**Methods:**

Between 2011 and 2014, 120 cases underwent outside-in ACL reconstruction. Forty-seven patients who had high TTT values were individually matched for age, sex, body mass index, and meniscus injury to a low TTT value group of 47 patients. The primary outcome was the occurrence of cyclops nodule formation or cyclopoid scar formation. All 94 patients were divided into 3 groups using surgical records and intra-operative video to enable a sub-analysis. The groups were a no-cyclops group, a cyclopoid group, and a cyclops group. Blood examinations, including TTT, and knee range of motion evaluations were performed before surgery, 3 months after surgery, and 1 year after surgery.

**Results:**

There were no differences in preoperative demographic data between the two groups. TTT values did not significantly influence cyclopoid scar formation (OR, 1.67; 95% CI, 0.62 to 4.66; *p* = 0.362). However, patients with cyclops nodule formation showed significantly higher TTT values than the control patients. (OR, 9.34; 95% CI, 1.94 to 90.3; *p* = 0.002). Knee extension loss was observed in the cyclopoid and cyclops groups 3 months after reconstruction. In the cyclops group, arthroscopic resection of the cyclops nodule was performed 3 months after reconstruction. Eventually, almost full range of motion was restored in all patients.

**Conclusions:**

High TTT values before ACL reconstruction were an indicator of cyclops nodule formation. Furthermore, cyclopoid scar formations may not be the result of an individual’s immune reaction but that of extension loss in the early post-reconstruction phase.

## Background

Cyclops nodule formation is a serious complication after anterior cruciate ligament (ACL) reconstruction, and it is characterized by loss of terminal knee extension due to proliferative fibrous nodule formation in the intercondylar notch [1]. The incidence of post-operative cyclops nodule formation ranges from 1.9 to 10.6% [2, 3], whereas the incidence of cyclops lesions without extension loss varies from 2.2 to 46.8% [4, 5]. This distinction of symptoms is due to 2 distinct types of cyclops lesions, a true cyclops nodule and a cyclopoid scar [6]. Although there are several hypotheses regarding the pathogenesis of cyclops nodule formation, including bone and cartilage residue in the joint following tibial tunnel drilling and preparation for graft passage [1, 6], repeated graft impingement on the notch [1], post-operative hamstring contracture [7], and narrowing of the femoral intercondylar notch [8], histologically, a cyclops nodule formation is composed of disorganized fibrous connective tissue with a central region of granulation tissue and newly formed vessels [1, 5, 6]. Cyclopoid scar formations are composed of a build-up of fibrous tissue showing elements of granulation tissue [6]. However, the same symptoms, along with similar arthroscopic and histologic findings, also occur in acute ACL injury without reconstruction [9, 10]. Therefore, the reparative processes occurring as an immune reaction of the vital tissue may be the main triggering factors for the process of cyclops nodule formation.

The thymol turbidity test (TTT) is a type of colloidal reaction test that reflects immunoglobulin M [11]. TTT is considered a marker of inflammatory conditions such as chronic hepatitis, chronic infection, or collagen disease [12, 13]. Before ACL reconstruction surgery, we routinely perform blood examination, including the TTT. By chance, we discovered that TTT results tended to be higher in patients with cyclops nodule formation, whereas there were no other blood examination abnormalities. To the best of our knowledge, a relationship between an immune reaction of the vital tissue and cyclops or cyclopoid development has been proposed, but not yet proven [6, 9]. In addition, there are no reports on blood examinations in patients with cyclops syndrome. The purpose of our study was to investigate whether an increase in TTT value was involved in the occurrence of cyclops nodule formation or cyclopoid scar formation. Furthermore, in order to investigate this in detail, a comparison between 3 groups (a no-cyclops group, a cyclopoid group, and a cyclops group) was performed using blood test results and knee range of motion measurements.

## Methods

### Study subjects

**Fig. 1:**
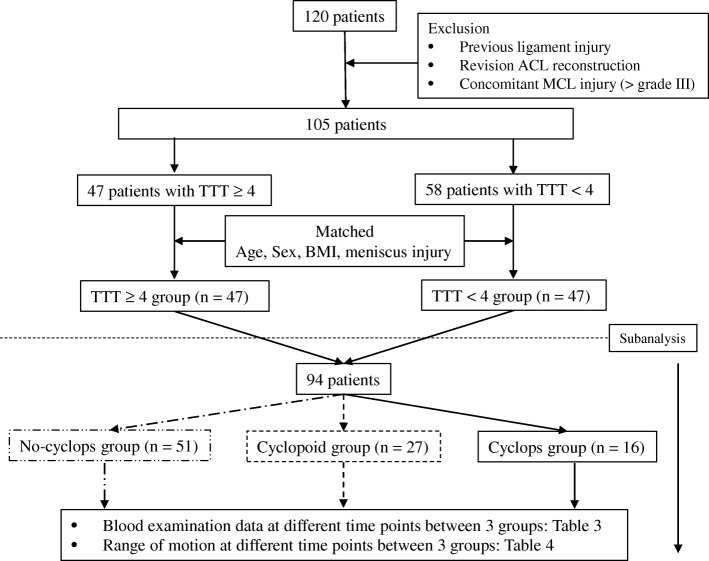
Flow diagram of patients screened and grouped. ACL, anterior cruciate ligament; MCL, medial collateral ligament; BMI; body mass index; TTT, thymol turbidity test

This retrospective study was performed with the approval of the institutional review board, and all patients signed the consent form drafted for the study. Between 2011 and 2014, 120 consecutive patients underwent outside-in ACL reconstruction [14] performed by two surgeons at our hospital. Exclusion criteria were patients who had previous ligament injury, and those who had a concomitant medial collateral ligament injury classified as greater than grade III. Patients who had undergone revision ACL reconstruction were also excluded. Finally, 47 patients with TTT ≥ 4 and 58 patients with TTT < 4 were included. The 47 patients in the TTT ≥ 4 group were matched for age, sex, and body mass index (BMI) with 47 patients in the TTT < 4 group (Fig. 1). In order to conduct case-control research, the research design was set as follows. The population included patients for whom final assessments could be made after reconstruction following an ACL tear. Exposure was defined as a TTT value ≥4 and control was defined as a TTT value < 4. Outcomes included the occurrence of cyclops nodule formation or cyclopoid scar formation. Cyclops and cyclopoid lesions were diagnosed using arthroscopic video based on a previous report [6].

**Fig. 2:**
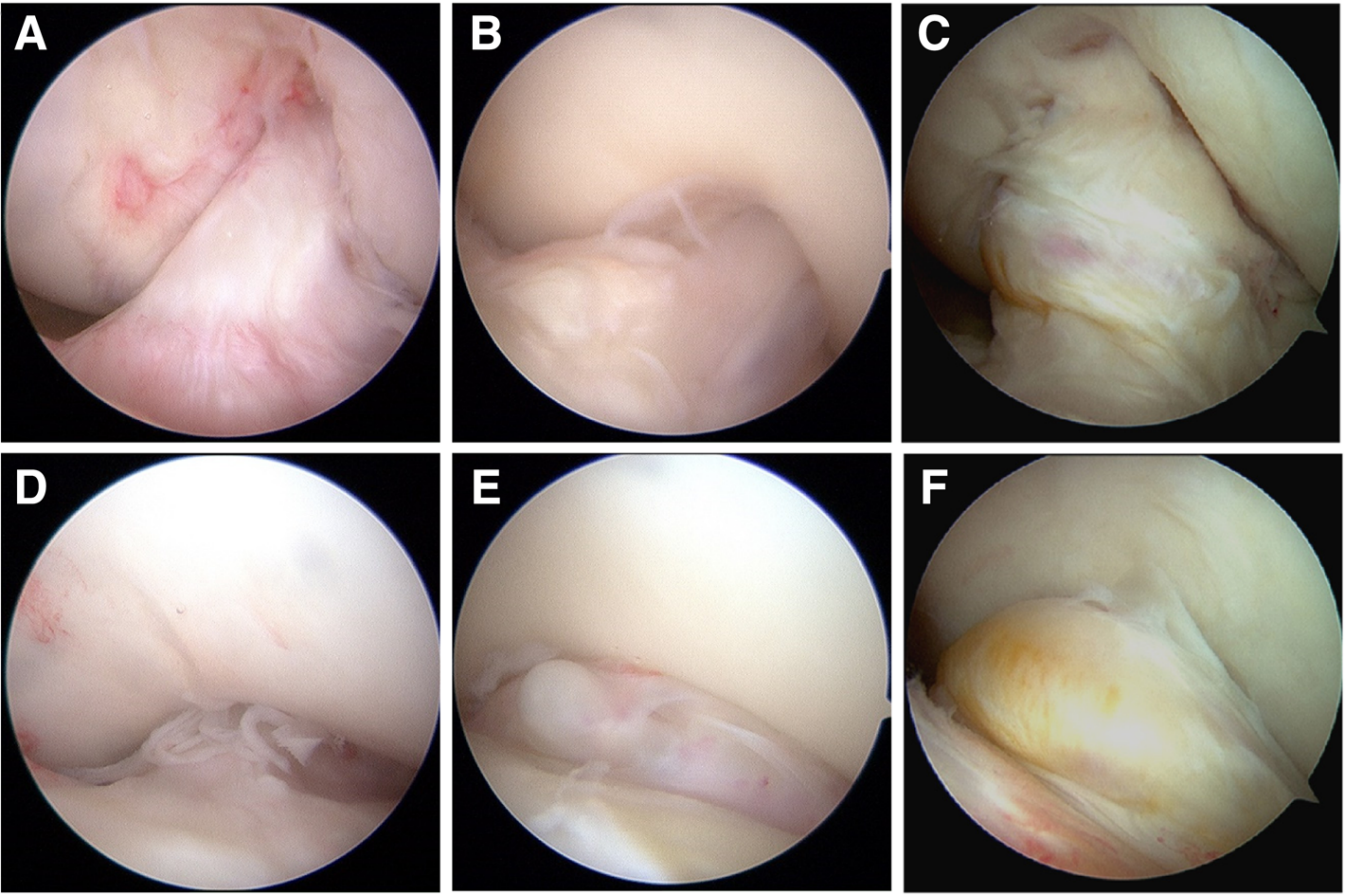
Arthroscopic findings during second-look arthroscopy after ACL reconstruction. Knee flexion position (**a**-**c**) and extension position (**d**-**f**) are shown. A patient without cyclops (**a**, **d**). A patient with a cyclopoid lesion (**b**, **e**). A patient with a cyclops lesion impinging on the intercondylar notch (**c**, **f**)

All 94 patients were divided into 3 groups using surgical records and intra-operative video to perform a sub-analysis (Fig. 2). These groups were a cyclops group (case) (*n* = 16), a no-cyclops group (control 1) (*n* = 51), and a cyclopoid group (control 2) (*n* = 27). In addition to the TTT, aspartate transaminase (AST), alanine transaminase (ALT), and C-reactive protein (CRP) levels were evaluated 1 week before reconstruction. The same inspection was performed after cyclops resection (3 months after reconstruction) and 1 week before second-look arthroscopy. The knee range of motion at 3 different time points was determined from clinical records.

### Surgical procedure

A double-bundle, outside-in, arthroscopic ACL reconstruction was performed in all patients, using the semitendinosus tendon (ST) and, if necessary, the gracilis tendon. The harvested tendons were double-looped over an Endobutton fixation device (Smith & Nephew, Andover, MA), with the distal ends anchored using a Krackow suture, thus recreating the anteromedial (AM) and posterolateral (PL) bundles of the ACL. To prevent elongation of the grafts, a continuous 30-s loading with 70 N was applied twice to the graft (70 N-1 min), and then the same loading was applied repeatedly (70 N-2 min) [15]. The femoral tunnel was created using an outside-in technique. The longitudinal linear resident’s ridge [16] and the posterior cartilage, used as landmarks for the ACL femoral footprint, were identified. Two 2.4-mm guide pins were then inserted separately from the outside into the ACL footprints behind the resident’s ridge and just anterior to the articular margin, using an anterolateral entry femoral aimer (Smith & Nephew). A 5.5-mm to 6.5-mm tunnel was then created for the AM and PL grafts by over-drilling via the guide pins. The autogenous tendon was harvested and transected into 2 double-looped grafts. Two Endobutton-CLs® (Smith & Nephew) were connected to the end of each loop graft. The appropriate graft length was determined from the length of the femoral tunnel to allow the introduction of sufficient graft materials (> 13 mm) into the bone tunnels. After creation of the femoral tunnel, the ACL tibial tunnel was created. The AM tunnel was created using the following intra-articular landmarks: just lateral to the medial intercondylar ridge and just posterior to the anterior ridge so as not to damage the lateral meniscus anterior insertion [17, 18]. The PL tunnel was created posterior to the AM tunnel, just lateral to the medial intercondylar ridge. In all cases, tibial fixation of the graft was performed using double-spike plates (Meira, Aichi, Japan), with the knee flexed at 20°, and an initial tension of 20 N was applied to the PL bundle and 30 N to the AM bundle. The tension in each bundle was measured independently using a tensiometer. Finally, we checked for impingement to the notch at full extension. In all cases, there was no impingement to the notch at full extension. Thus, femoral notchplasty was not performed in all cases.

### Second-look arthroscopic examination, clinical evaluations, and post-operative management

Second-look arthroscopy was performed approximately 1 year after reconstruction for the removal of the 2 double-spike plates, fixed with screws into the tibia, which were used for tibial fixation of the ACL graft. Knee range of motion was evaluated with a goniometer before reconstruction, 3 months after reconstruction, and after second-look arthroscopy. Extension loss was measured in degrees and compared with the normal contralateral extremity. For post-operative rehabilitation, knees without associated meniscal tears were maintained in a brace for 1 week, and knees with meniscus sutures were immobilized for 2 weeks. After immobilization, all patients followed the same rehabilitation protocol including isometric exercises, range of motion exercises, and proprioceptive rehabilitation.

### Blood examination

Blood examination, including TTT, was performed in all patients automatically using a reagent (Clinimate TTT reagent, Sekisui Medical, Japan) that does not require adjustment. This reagent can be used in an automatic analyzer. The reference standard range was set to 4 McLagan units or less.

### Statistical analysis

Descriptive data were presented as the mean ± standard deviation (SD). We first performed a Fisher’s exact test to obtain odds ratios (ORs) of the occurrence of cyclops nodule formation and cyclopoid scar formation in the control and exposure groups. An independent-samples Student’s *t* test was used to compare group differences for normally distributed variables. The Mann-Whitney *U* test was used for non-normally distributed variables and a one-way analysis of variance with the Fisher protected least significant difference test for post hoc multiple comparisons. All analyses were performed using SPSS 11.0. Statistical significance was set at *p* < 0.05, a priori.

## Results

As mentioned above, 47 patients were included in each group in this retrospective study. There were no differences in preoperative patient characteristics between the two groups (Table 1). TTT values did not significantly influence cyclopoid scar formation (OR, 1.67; 95% CI, 0.62 to 4.66; *p* = 0.362). However, patients with cyclops nodule formation showed significantly higher TTT values than the control patients. (OR, 9.34; 95% CI, 1.94 to 90.3; *p* = 0.002) (Table 2).

**Table 1:** Preoperative patient characteristics

	TTT ≥ 4 (*n* = 47)	TTT < 4 (*n =* 47)	*P* value
Mean age, years	24.0 ± 7.4	24.0 ± 6.4	0.989
Gender (Male/Female)	26/21	26/21	1.000
BMI, kg/m^2^	23.7 ± 3.3	23.2 ± 3.0	0.488
Meniscal injury, n, %	33 (70.2%)	25 (53.2%)	0.137

Data are expressed as the mean ± SD

*TTT* thymol turbidity test, *BMI* body mass index

**Table 2:** Odds of cyclops nodule and cyclopoid scar following TTT value

	TTT ≥ 4 (*n =* 47)	TTT < 4 (*n =* 47)	OR (95% CI)	*P* value
Cyclops nodule, n, %	14 (29.8%)	2 (4.26%)	9.34 (1.94–90.3)	0.002*
Cyclopoid scar, n, %	16 (34.0%)	11 (23.4%)	1.67 (0.62–4.66)	0.362

Data are expressed as the mean ± SD

*Statistically significant difference (*P* < 0.05)

*TTT* thymol turbidity test

Cyclops nodule formation was found in 16 of the 94 patients (14.9%) and cyclopoid lesions were found in 27 patients (28.7%) during second-look arthroscopy. Blood examination data before ACL reconstruction showed that the cyclops group (case) had a significant highly TTT value compared to the no-cyclops (control 1) and cyclopoid group (control 2) (6.3 ± 3.6, 3.3 ± 2.0, and 3.8 ± 2.4, respectively; *p* < 0.05) (Table 3). There was no difference in TTT values in the no-cyclops group (control 1) and the cyclopoid group (control 2). When comparing the different time point blood examinations, TTT values were significantly lower after resection of the cyclops lesion (3 months after reconstruction) and before second-look arthroscopy compared to before reconstruction. After cyclops nodule resection, TTT values increased slightly until before second-look arthroscopy, but did not return to pre-reconstruction TTT values.

**Table 3:** Blood examination data at different time points between 3 groups

Blood examination (mean value)	Control 1 (*n =* 51)	Control 2 (*n =* 27)	Case (*n* = 16)	F value
Before ACL reconstruction				
TTT	3.3 ± 2.0	3.8 ± 2.4	6.3 ± 3.6^a^	8.86
AST	19.2 ± 3.7	19.1 ± 4.9	20.9 ± 5.8	2.25
ALT	20.6 ± 6.3	20.6 ± 8.3	22.7 ± 13.1	3.65
CRP	0.1 ± 0.1	0.1 ± 0.2	0.1 ± 0.1	0.88
3 months after reconstruction (after cyclops resection)				
TTT	–	–	3.5 ± 1.3^b^	
AST	–	–	20.4 ± 4.9	
ALT	–	–	22.5 ± 10.5	
CRP	–	–	0.10 ± 0.1	
Before second-look arthroscopy				
TTT	3.4 ± 1.9	3.4 ± 1.5	4.5 ± 1.2^a/b^	9.86
AST	21.2 ± 4.5	20.4 ± 3.9	21.6 ± 6.7	2.21
ALT	20.5 ± 5.8	19.1 ± 8.3	22.7 ± 11	3.75
CRP	0.1 ± 0.5	0.2 ± 0.6	0.1 ± 0.3	1.25

Data are expressed as the mean ± SD

*ACL* anterior cruciate ligament, *TTT* thymol turbidity test, *AST* aspartate transaminase, *ALT* alanine transaminase, *CRP* C-reactive protein

^a^*P* < 0.05 when compared with control 1 and control 2 group, using post hoc multiple comparisons

^b^*P* < 0.05 when compared with before reconstruction, using Student’s *t* test

Range of motion was compared in the 3 groups (Table 4). There was no significant difference in the 3 groups before reconstruction. Extension loss was observed in the cyclopoid (control 2) and cyclops groups (case) 3 months after reconstruction. In addition, knee flexion was also restricted in the cyclops group (case) compared to that in the no-cyclops group (control 1). In the cyclops group (case), arthroscopic resection of the cyclops nodule was performed 3 months after reconstruction. Eventually, almost full range of motion was restored in all patients and there were no dissatisfied patients.

**Table 4:** Range of motion at different time points

Range of motion	Control 1 (*n =* 51)	Control 2 (*n =* 27)	Case (*n =* 16)	F value
Before reconstruction				
Extension (°)	1.3 ± 2.6	1.0 ± 2.8	1.2 ± 2.6	2.08
Flexion (°)	135.5 ± 9.8	136.2 ± 6.8	135.2 ± 8.5	1.37
3 months after reconstruction				
Extension (°)	1.3 ± 2.6	−6.9 ± 3.8*	− 10.2 ± 4.8*	18.9
Flexion (°)	130.5 ± 7.2	131.2 ± 5.8	118 ± 5.5*	12.5
After second-look arthroscopy				
Extension (°)	1.3 ± 2.6	− 1.2 ± 1.8	−1.8 ± 3.2	2.23
Flexion (°)	135.5 ± 9.8	136.2 ± 6.8	134.0 ± 5.5	1.28

Data are expressed as the mean ± SD

*Statistically significant difference (*P* < 0.05)

## Discussion

The most important finding of this study was that high TTT values before ACL reconstruction may be a potential risk factor for developing cyclops nodule formation. Many researchers have reported that the cause of cyclops syndrome (due to an impinged cyclops nodule) is multi-factorial, and therefore, the pathological condition is not completely understood [1, 7, 8]. In fact, although surgical procedures corresponding to the causative disease condition have been reported [19, 20], surgical procedures still fail to prevent the occurrence of cyclops lesions. We believe the cyclops nodule formation that occurs after surgery is related to the individual’s immune response. This is because the patterns observed during cyclops nodule formation cannot be explained by the potential causes that have been reported so far. The clinical problem of cyclops syndrome (due to an impinged cyclops nodule) is to cause loss of irreversible knee extension that does not improve without surgery. Furthermore, nodule formation is considered to be completed by about 6 weeks after surgery [7]. On the other hand, it has been reported that resection of cyclops nodule formation improves symptoms without recurrence. This suggests that acute vital tissue reactions occurring after ACL injury or in the early phase after reconstruction may be involved in development of cyclops nodules. The continuous contact between the graft and intercondylar notch may produce an irritating stimulus, which may induce an inflammatory response with the production of granulation tissue, which would be transformed into fibrocartilaginous and cartilaginous tissue [21]. However, because all knees in our cohort achieved full extension at the end of surgery, failure to regain full extension may be due the wound healing process after surgery. Intra- or post-operative factors may promote the process, but they are not the key factors. Injury to the ligament and the reparative processes occurring as a result of vital tissue reactions are the main triggering factors for cyclops nodule formation [9]. The response of living tissue in this reparative process varies from individual to individual, and it is possible that an immune response is involved.

We discovered that TTT values associated with the risk of developing cyclops nodule formation following ACL reconstruction. TTT was reported as an indicator of hepatic injury in the mid-twentieth century [13]. Basically, this examination reflects a decrease in serum albumin and an increase in globulin. γ globulin has a tendency to precipitate, and this increases the amount of sedimentation; however, if hydrophilic albumin increases, γ globulin does not precipitate. The TTT value is relatively high even with lower albumin levels associated with chronic hepatitis and chronic inflammation. Although, we did evaluate AST, ALT, and CRP, and there were no differences at the different time points (before reconstruction, at 3 months, and 1 year after reconstruction) (Table 3). Previous reports have shown that TTT is associated with immune reaction [11, 21, 22]. Our findings are clinically relevant, since the pathophysiological effect of the presence of a cyclops nodule formation is not fully understood to date.

We performed cyclops resection in the cyclops group 3 months after reconstruction. Interestingly, the high pre-operative TTT values were reduced following cyclops resection. Furthermore, the TTT values did not return to the pre-reconstruction values before second-look arthroscopy, but they were higher than the no-cyclops group (Table 3). It may be suggested that resection of the cyclops using the arthroscope suppressed the reaction that occurs in the body of a living organism when the body rejects something. Furthermore, the gradually increasing TTT values after nodule resection may indicate that the patients who developed cyclops nodule formation originally had high immunoglobulin levels.

There was no difference in TTT values in the group that developed cyclopoids and the no-cyclops group. The cyclopoid is the displaced portion of the ACL with an angulated fold at the anterior end, giving it a tongue-like appearance [1, 23]. Histologically, cyclopoid scar formations are made up of fibrous tissue, showing elements of granulation tissue [6]. Similar to these reports, the cyclopoid scars observed during second-look arthroscopy in our study were recognized as soft, scar-like tissue in front of the ACL graft impinging on the intercondylar region during knee extension (Fig. 2). Regarding range of motion in the cyclopoid group, extension loss was observed at 3 months (Table 4), and 10 patients showed extension loss of more than 10 degrees 3 months after reconstruction. However, due to no palpable “clunk” with terminal extension, we did not perform surgery on the cyclopoid group. As a result, in the cyclopoid group, range of motion recovered to that of the contralateral knee 1 year after reconstruction in all cases. Extension loss in the early phase after reconstruction prevents closure of the intercondylar notch and allows local organization of the post-operative hemarthrosis [24]. Soft tissues such as cyclopoid scars may not be the result of an individual’s immune reaction, but due to extension loss in the early post-reconstruction phase.

Given that this was a retrospective case control study, we did not examine other joint abnormalities. Given that flexion was also limited in the cyclops group, we further believe that the development of this nodulous scar formation is merely the expression of a generalized inclination to fibrotic healing. We showed that the occurrence of cyclops nodule formation may be involved in the response of living tissue, which is a potential factor that varies between individuals. Given this, low-dose steroids administered for the treatment of arthrofibrosis may be effective for patients with high TTT values.

### Limitations

Although we have shown that the development of cyclops nodule formation depends on TTT values, TTT values are said to vary depending on the amount of immunoglobulin; however, we did not evaluate antibody-producing lymphocytes or antigen-presenting dendritic cells. Further evaluations using immunostaining of cyclops tissue are necessary. In addition, further studies are required to investigate the secretion of cytokines, growth factors, chemokines, inflammatory mediators, and matrix molecules and proteins that contribute to motility, proliferation, and differentiation of fibroblasts and myofibroblasts involved in the growth phase during wound healing.

## Conclusions

High TTT values before ACL reconstruction were a potential risk factor for developing cyclops nodule formation. Furthermore, cyclopoid scar formations may not be the result of an individual’s immune reaction, but due to extension loss in the early post-reconstruction phase.

## Abbreviations

ACL: Anterior cruciate ligament
ALT: Alanine transaminase
AM: Anteromedial
AST: Aspartate transaminase
BMI: Body mass index
CRP: C-reactive protein
CT: Computed tomography
MR: Magnetic resonance
PL: Posterolateral
ST: Semitendinosus tendon
TTT: Thymol turbidity test
